# Interference between rhinovirus and influenza A virus: a clinical data analysis and experimental infection study

**DOI:** 10.1016/s2666-5247(20)30114-2

**Published:** 2020-09-05

**Authors:** Anchi Wu, Valia T Mihaylova, Marie L Landry, Ellen F Foxman

**Affiliations:** Department of Laboratory Medicine (A Wu BSE, V T Mihaylova PhD, Prof M L Landry MD, Prof E F Foxman MD), Department of Internal Medicine (Prof M L Landry), and Department of Immunobiology (A Wu, Prof E F Foxman), Yale University School of Medicine, New Haven, CT, USA

## Abstract

**Background:**

During the 2009 pandemic of an emerging influenza A virus (IAV; H1N1pdm09), data from several European countries indicated that the spread of the virus might have been interrupted by the annual autumn rhinovirus epidemic. We aimed to investigate viral interference between rhinovirus and IAV with use of clinical data and an experimental model.

**Methods:**

We did a clinical data analysis and experimental infection study to investigate the co-occurrence of rhinovirus and IAV in respiratory specimens from adults (≥21 years) tested with a multiplex PCR panel at Yale-New Haven Hospital (CT, USA) over three consecutive winter seasons (Nov 1 to March 1, 2016–17, 2017–18, and 2018–19). We compared observed versus expected co-detections using data extracted from the Epic Systems electronic medical record system. To assess how rhinovirus infection affects subsequent IAV infection, we inoculated differentiated primary human airway epithelial cultures with rhinovirus (HRV-01A; multiplicity of infection [MOI] 0·1) or did mock infection. On day 3 post-infection, we inoculated the same cultures with IAV (H1N1 green fluorescent protein [GFP] reporter virus or H1N1pdm09; MOI 0·1). We used reverse transcription quantitative PCR or microscopy to quantify host cell mRNAs for interferon-stimulated genes (ISGs) on day 3 after rhinovirus or mock infection and IAV RNA on days 4, 5, or 6 after rhinovirus or mock infection. We also did sequential infection studies in the presence of BX795 (6 μM), to inhibit the interferon response. We compared ISG expression and IAV RNA and expression of GFP by IAV reporter virus.

**Findings:**

Between July 1, 2016, and June 30, 2019, examination of 8284 respiratory samples positive for either rhinovirus (n=3821) or IAV (n=4463) by any test method was used to establish Nov 1 to March 1 as the period of peak virus co-circulation. After filtering for samples within this time frame meeting the inclusion criteria (n=13 707), there were 989 (7·2%) rhinovirus and 922 (6·7%) IAV detections, with a significantly lower than expected odds of co-detection (odds ratio 0·16, 95% CI 0·09–0·28). Rhinovirus infection of cell cultures induced ISG expression and protected against IAV infection 3 days later, resulting in an approximate 50 000-fold decrease in IAV H1N1pdm09 viral RNA on day 5 post-rhinovirus inoculation. Blocking the interferon response restored IAV replication following rhinovirus infection.

**Interpretation:**

These findings show that one respiratory virus can block infection with another through stimulation of antiviral defences in the airway mucosa, supporting the idea that interference from rhinovirus disrupted the 2009 IAV pandemic in Europe. These results indicate that viral interference can potentially affect the course of an epidemic, and this possibility should be considered when designing interventions for seasonal influenza epidemics and the ongoing COVID-19 pandemic.

**Funding:**

National Institutes of Health, National Institute of General Medical Sciences, and the Yale Department of Laboratory Medicine.

## Introduction

The COVID-19 pandemic has placed new urgency on examining the underlying mechanisms that influence the epidemic spread of respiratory viruses. One proposed mechanism is viral interference, a phenomenon in which infection with one virus provides transient protection against infection with other related or unrelated viruses.^1^

In the past decade, advances in genome-based virus detection have markedly improved the ability to diagnose respiratory virus infections. As these methods become more widespread, accumulating test results indicate that viral interference could be shaping human respiratory virus epidemics. Attention became focused on this idea during the 2009 pandemic of an emerging influenza A virus (IAV), when data from several European countries indicated that the annual autumn rhinovirus epidemic interrupted and delayed transmission of the emerging influenza virus.^2–4^ Since 2009, analyses of co-detections of common respiratory viruses, including rhinovirus and IAV or rhinovirus and respiratory syncytial virus (RSV), have shown that co-detections are significantly lower than would be expected by chance alone, also supporting the viral interference hypothesis.^3,5–10^ Another important observation was made in a small study of the live attenuated intranasal influenza vaccine, in which investigators observed decreased replication of the influenza vaccine strain in children who tested positive for another respiratory virus at the time of vaccine exposure.^11^ In a mouse model, previous rhinovirus exposure attenuated IAV infection, with the caveat that rhinovirus does not replicate in mice.^12^ Increasing use of multiplex testing for respiratory viruses over the past decade, coupled with advances in electronic medical record keeping and bioinformatics, offers the opportunity to compare observed and expected viral co-detection rates on a much larger scale than previously possible to further evaluate evidence for viral interference.

A challenge of evaluating viral interference based on clinical observations alone is absence of information for causality. Factors other than viral interference could contribute to low virus co-detection rates, such as differences in virus seasonality based on environmental factors or differences in virus host range (eg, viruses preferentially infect different age groups). In this study, we addressed possible confounders by limiting our analysis to an age group and time window for which detection rates for both viruses were approximately equal in our patient population. To address causality, we sequentially infected differentiated primary human airway epithelial cells cultured at air–liquid interface. Here, we report the results of a rhinovirus–IAV co-detection analysis with use of patient data and the results of experimental infection studies, both designed to evaluate evidence for interference between rhinovirus and IAV.

## Methods

### Study design

We did a clinical data analysis to investigate the co-occurrence of rhinovirus and IAV at Yale-New Haven Hospital (CT, USA) and an experimental infection study to investigate interference between the two viruses. For the clinical data analysis, to establish a time frame and age group in which rhinovirus and IAV were co-circulating in patients in our health-care system from 2016 to 2019, we examined data from all testing methods and all age groups with a test result summary generated by the Yale-New Haven Hospital clinical virology laboratory. Test methods are described in the appendix (pp 2–4). On the basis of these data, test results for adults (≥21 years) from Nov 1 to March 1, 2016–17, 2017–18, and 2018–19, were selected for analysis since the majority of tests were for adults (88%), and detections of rhinovirus and IAV were approximately equal in this age group.

Co-detection analysis was done only on samples that had been tested with the complete laboratory-developed Yale-New Haven Hospital respiratory virus PCR panel for the following ten viruses: rhinovirus; IAV; influenza B virus (IBV); RSV A and B; parainfluenza viruses 1, 2, and 3; human metapneumovirus; and adenovirus, as described previously,^13^ with rhinovirus primers updated in March, 2018^14^ (appendix p 4). Test results were retrieved from the Yale-New Haven Hospital Epic Systems electronic medical record system using a custom report created by the Yale Joint Data Analytics Team and exported into a Microsoft Excel spreadsheet on an encrypted computer. Data retrieved were: patient age and sex, date of testing, tests results for each virus, and test platform. Patient medical record number was retrieved but was deleted and replaced with an anonymised sample number before analysis. Data were filtered to include adults only (≥21 years), and to exclude repeat tests on the same patient within the same week (appendix p 7).

The study protocol was reviewed and approved by the Yale Human Investigation Committee and was determined to not require specific patient consent or institutional review board review.

### Procedures

We established a co-infection model using primary human airway epithelial cells differentiated at air–liquid interface to form organoid cultures that recapitulate the mucosal surface in vivo. The cells from healthy adult donors were obtained commercially (Lonza, Walkersville, MD, USA) and cultured according to the manufacturer’s instructions using reduced hydrocortisone (Stem Cell Technologies, Vancouver, BC, Canada). Cells were allowed to differentiate for 4 weeks, by which time they displayed beating cilia and mucus production. Preliminary experiments established that when infected at a multiplicity of infection (MOI) of 0·1, these cultures supported robust replication of both rhinovirus and IAV and survived each infection, making sequential infection experiments possible and justifying our choice of this MOI for co-infection experiments.

For infection, the apical surface of each primary human airway epithelial cell culture was washed with 200 μL of warm phosphate buffered saline (PBS; Sigma-Aldrich, Burlington, MA, USA), then cultures were inoculated with rhinovirus 1A (HRV-01A; VR-481; American Type Culture Collection [ATCC], Manassas, VA, USA) MOI 0·1 per well in 200 μL PBS with 0·1% bovine serum albumin (AmericanBio, Natick, MA, USA) for 1 h at 35°C, after which time the inoculum was removed, the apical surface was rinsed with PBS, and basolateral medium was replaced with fresh medium. Cells were incubated at 35°C for 3, 24, 48, or 72 h to establish infection kinetics. For the sequential infection model, basolateral medium was supplemented with 150 μL fresh medium on day 3 and mock inoculation or inoculation with IAV (MOI 0·1) was done with the same procedures as for rhinovirus 1A. Cells were inoculated with a previously described H1N1 IAV that expresses a green fluorescent protein (GFP) reporter during replication (PR8-GFP), generously shared by the García-Sastre Laboratory (Icahn School of Medicine at Mount Sinai, New York, NY, USA).^15^ We assessed the effect of previous exposure to rhinovirus on host response and IAV replication using reverse-transcription quantitative PCR (RT-qPCR) for host cell mRNAs for ISG15, RSAD2 (Viperin), MX1, and IFITM3 on day 3 post-rhinovirus infection, and RT-qPCR for IAV RNA on days 4 and 5 post-rhinovirus infection (24 and 48 h post-IAV infection, respectively). We also quantitated GFP-expressing cells using confocal fluorescence microscopy on day 4 post-rhinovirus infection (24 h post-IAV infection).

To evaluate the effect of rhinovirus on subsequent infection with IAV H1N1pdm09 (strain A/California/07/2009; ATCC VR-1894), we infected differentiated airway epithelial cells with each virus individually and sequentially, then examined the time course of viral amplification and interferon-stimulated gene (ISG) induction (by RT-qPCR), using the same infection procedures as for IAV PR8-GFP. Cells were incubated at 35°C for 3, 24, 48, or 72 h to establish IAV H1N1pdm09 infection kinetics. In sequential infection experiments, cells were collected at day 4 post-rhinovirus infection (24 h after mock or IAV infection) to assess the effect of sequential infection on ISG induction and on day 4, 5, or 6 post rhinovirus infection (24–72 h post-IAV infection) to assess IAV RNA expression.

To examine the effect of the interferon response on IAV H1N1pdm09 replication, we added 1000 U/mL of recombinant interferon beta (PBL Assay Science, Piscataway, NJ, USA) in the basolateral medium 18 h before inoculation with IAV (MOI 0·1). We assessed the effect on viral replication and induction of ISGs by RT-qPCR 24 h after infection with IAV.

To formally test whether previous exposure to rhinovirus inhibits IAV replication through activation of the host cell interferon response, we did sequential infection studies in the presence of BX795 (Sigma Aldrich, Burlington, MA, USA), a drug that blocks innate immune signalling required for the interferon response.^16^ BX795 was added to the basolateral medium 18 h before inoculation with rhinovirus at a concentration of 6 μM, and was maintained at the same concentration throughout the experiment. Cultures were collected for RNA isolation and RT-qPCR to measure induction of ISGs at day 3 post-rhinovirus infection, or collected at day 5 post-rhinovirus infection (48 h post-IAV infection) for RT-qPCR to quantitate rhinovirus and IAV viral RNA. Details of RT-qPCR and confocal fluorescence microscopy are described in the appendix (pp 2–3). Results shown are representative of at least three independent experiments.

### Statistical analysis

To evaluate the possibility of an interaction between rhinovirus and IAV, we compared the observed and expected co-detections of these two viruses using the patient test results meeting the inclusion criteria. First, we counted observed instances of single infections and co-infections for pair combinations of rhinovirus, IAV, IBV, RSV, parainfluenza virus, human metapneumovirus, and adenovirus. Next, we estimated the expected number of co-infections in the absence of interference for all virus pairs. Expected co-detection number was defined as the product of the incidence of virus 1 and the incidence of virus 2 multiplied by the total sample size. Next, we used χ^2^ or Fisher’s exact test (with a significance threshold of p<0·05) to assess whether there was a significant difference between the observed co-detections and expected co-detections in the absence of interference, using Python, version 3.7.3. We also calculated the odds ratios (OR) and corresponding 95% CIs for co-detection of each virus pair. Further details of these statistical tests are described in the appendix (p 3). To facilitate similar analyses by other investigators, we created a web tool to generate these statistics from a two by two contingency table, with the option to share data.

For experimental data, GraphPad Prism, version 8.4.2 was used for two-tailed *t* tests for pairwise comparison between conditions, and for two-way ANOVA to compare time series.

### Role of the funding source

The funder of the study had no role in study design, data collection, data analysis, data interpretation, or writing of the report. The corresponding author had full access to all the data in the study and had final responsibility for the decision to submit for publication.

## Results

**Figure 1:**
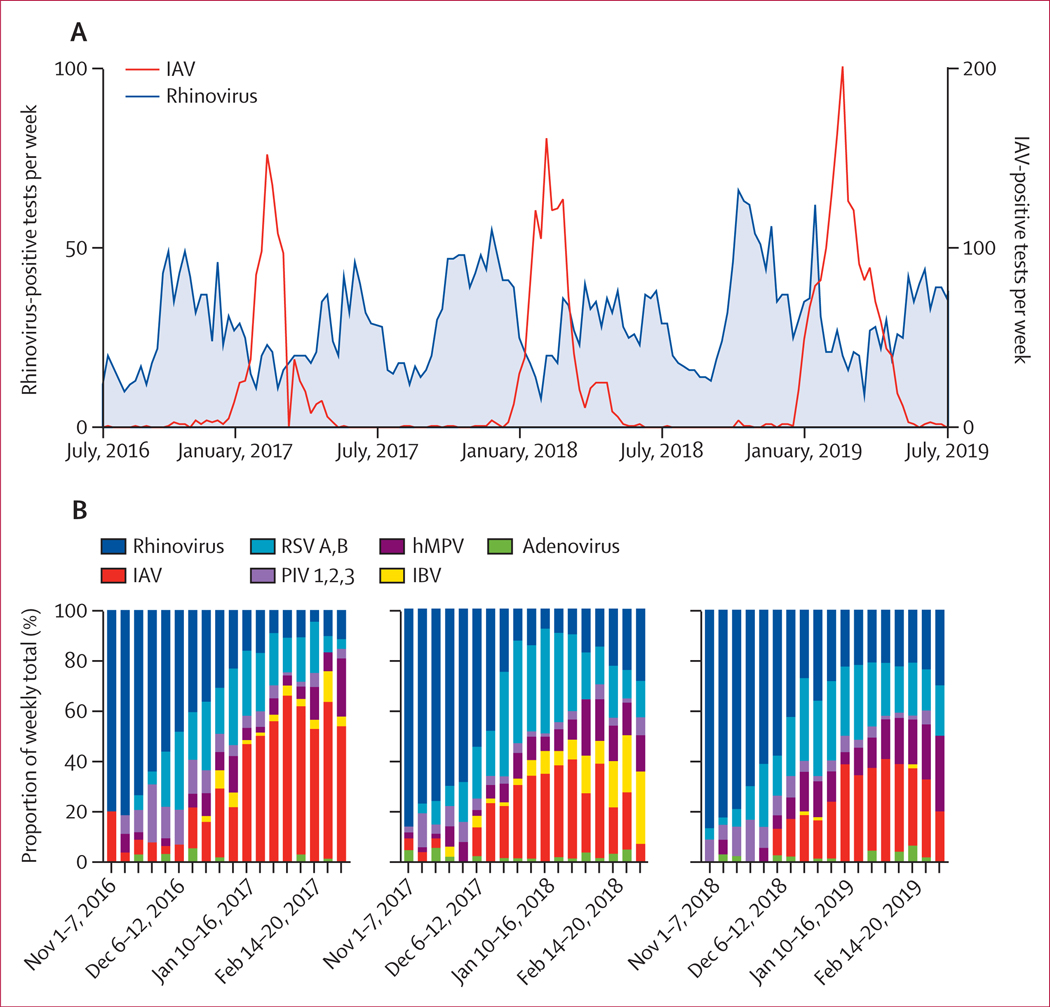
Virus detections by week, July, 2016, to June, 2019 (A) Total number of rhinovirus-positive and IAV-positive respiratory samples by week from July 1, 2016 (week 26), to June 30, 2019 (week 25). (B) Relative contribution of each virus to the total virus positive tests per week at Yale-New Haven Hospital for samples meeting the co-detection analysis inclusion criteria. IAV=influenza A virus. RSV=respiratory syncytial virus. IBV=influenza B virus. PIV=parainfluenza virus. hMPV=human metapneumovirus.

Between July 1, 2016, and June 30, 2019, 8284 respiratory samples tested at the Yale-New Haven Hospital clinical virology laboratory were positive for rhinovirus (n=3821) or IAV (n=4463) by all test methods, which included rapid influenza detection methods, direct fluorescent antigen detection, and PCR for a panel of respiratory viruses (appendix p 4). We observed seasonality consistent with other studies,^17,18^ with wide peaks of rhinovirus positive samples each autumn and spring, and a narrower peak of IAV positive samples between the rhinovirus peaks each winter (figure 1A). Peak rhinovirus and influenza co-circulation occurred from Nov 1 to March 1 each year (figure 1A). Next, we focused only on samples tested with the complete Yale-New Haven Hospital respiratory virus PCR panel during the Nov 1 to March 1 time frames, including data from 2016–17, 2017–18, and 2018–19. Before filtering, 15 940 test results were available for the respiratory virus PCR panel, with most tests done on adults aged 21 years or older (13 973 [87·7%]). There were roughly equal numbers of rhinovirus and IAV detections in adults and a slight female bias in both the number of tests done overall and the number of positive tests for rhinovirus and IAV (appendix p 6). After filtering to include adults only (≥21 years) and to remove repeat tests on a given patient within the same week, 13 707 results were available for co-detection analysis (appendix p 7). Rhinovirus and IAV results meeting the inclusion criteria mirrored seasonal trends in the larger sample dataset, with rhinovirus incidence declining and IAV rising between Nov 1 and March 1 each year (figure 1B). Overall, the number of rhinovirus and IAV positive samples after filtering was roughly equal, with 989 (7·2%) detections for rhinovirus and 922 (6·7%) detections for IAV (appendix p 5).

Rhinovirus and IAV showed a significantly lower than expected rate of co-occurrence during months of peak co-circulation. Co-detection analysis on test results meeting the inclusion criteria revealed a significant negative association between rhinovirus detection and IAV detection, with an OR of 0·16 (95% CI 0·09–0·28; table). The predicted number of co-detections for rhinovirus and IAV in the absence of any interaction was 67, but only 12 co-detections were observed (χ^2^ p value=1·08 × 10^−12^). In addition to our primary analysis of rhinovirus and IAV, secondary analyses comparing observed and expected co-detections among other virus pairs using test results meeting the inclusion criteria, based on two different statistical tests, revealed significant negative associations between rhinovirus and RSV, human metapneumovirus, and IBV; between IAV and RSV, human metapneumovirus, and parainfluenza virus; and between RSV and human metapneumovirus, and parainfluenza virus, as shown in the table.

**Figure 2:**
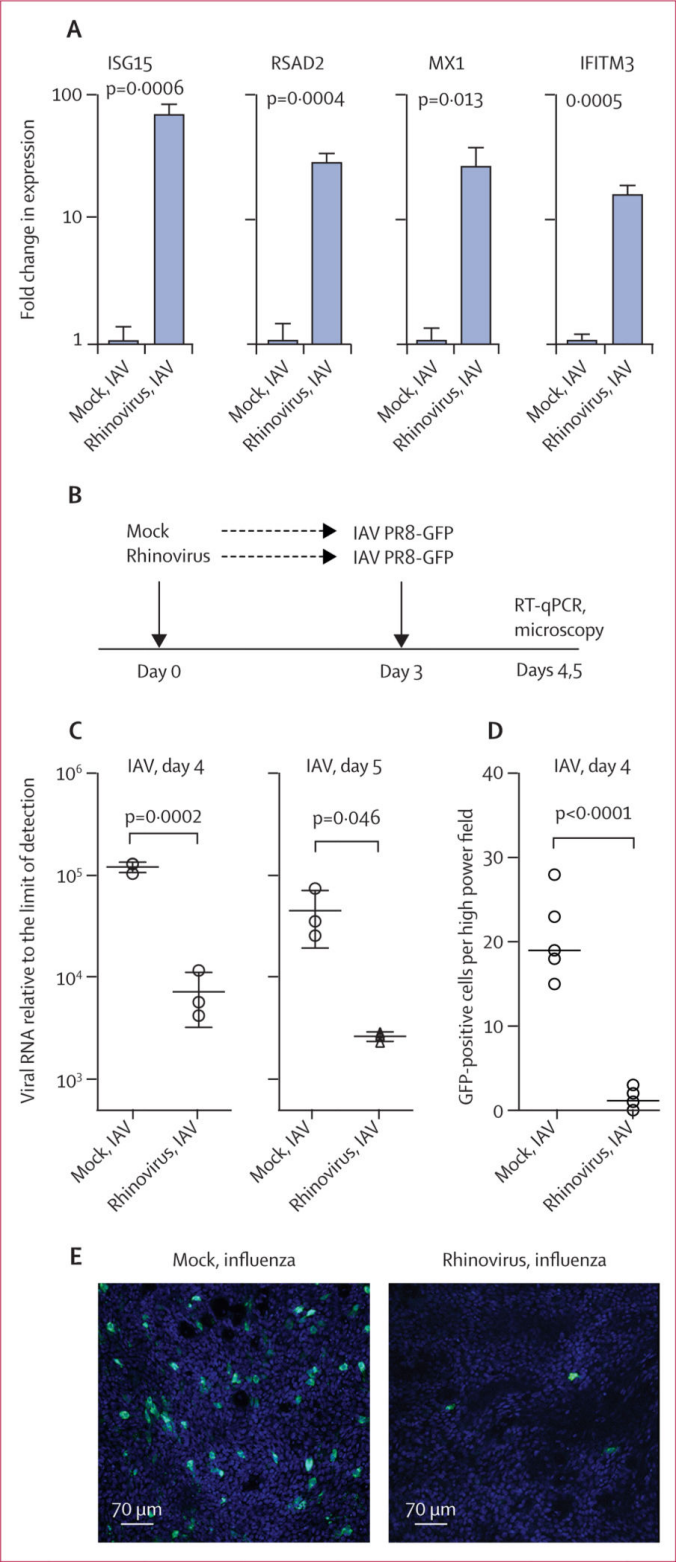
Effect of rhinovirus infection on epithelial gene expression and IAV infection (A) Interferon-stimulated gene mRNA expression in primary airway epithelial cultures 3 days post-infection with HRV-01A. Bars show fold change from mean expression in mock-treated cells, with values normalised to *HPRT*. (B) Design of sequential infection experiment. (C) Amount of viral RNA for IAV PR8-GFP on days 4 and 5 (24 h and 48 h post-IAV infection), graphed as fold change from limit of detection. (D) Number of GFP positive cells per HPF of seven different fields per condition, using 4·6 μm thick optical sections. (E) Projections of 17 μm thick optical sections of epithelial cultures, 24 h after infection with IAV PR8-GFP, with or without previous inoculation with rhinovirus. Scale bar=70 μm. Graphs show mean and SD of at least three biological replicates per condition. Results are representative of at least three independent experiments using primary cells from different healthy adult donors. IAV=influenza A virus. GFP=green fluorescent protein. HPF=high power field.

Infection of differentiated human airway epithelial cultures with HRV-01A (MOI 0·1) resulted in robust viral replication during the first 24–48 h, plateauing around day 3 post-infection (appendix p 8). At this timepoint, rhinovirus-infected cultures showed significant induction of mRNAs characteristic of the antiviral interferon response, including four ISGs that have been shown to encode effectors that block IAV infection (figure 2A, appendix p 8).^19^ Cultures inoculated with rhinovirus and then infected with a previously described H1N1 IAV GFP reporter virus (IAV PR8-GFP)^15^ at 3 days post-rhinovirus infection showed significant inhibition of IAV replication, as indicated by a more than 15-times reduction in IAV viral load by RT-qPCR at both 24 h and 48 h post-IAV infection (figure 2C). Imaging PR8-GFP-infected cultures with confocal fluorescence microscopy revealed a striking reduction in the number of GFP-positive cells in cultures with previous rhinovirus infection (figure 2D, 2E; videos 1, 2). Taken together, these findings show that rhinovirus infection induces an antiviral response in the human airway epithelium that persists at day 3 post-infection, and that rhinovirus infection interferes with subsequent IAV infection.

**Figure 3:**
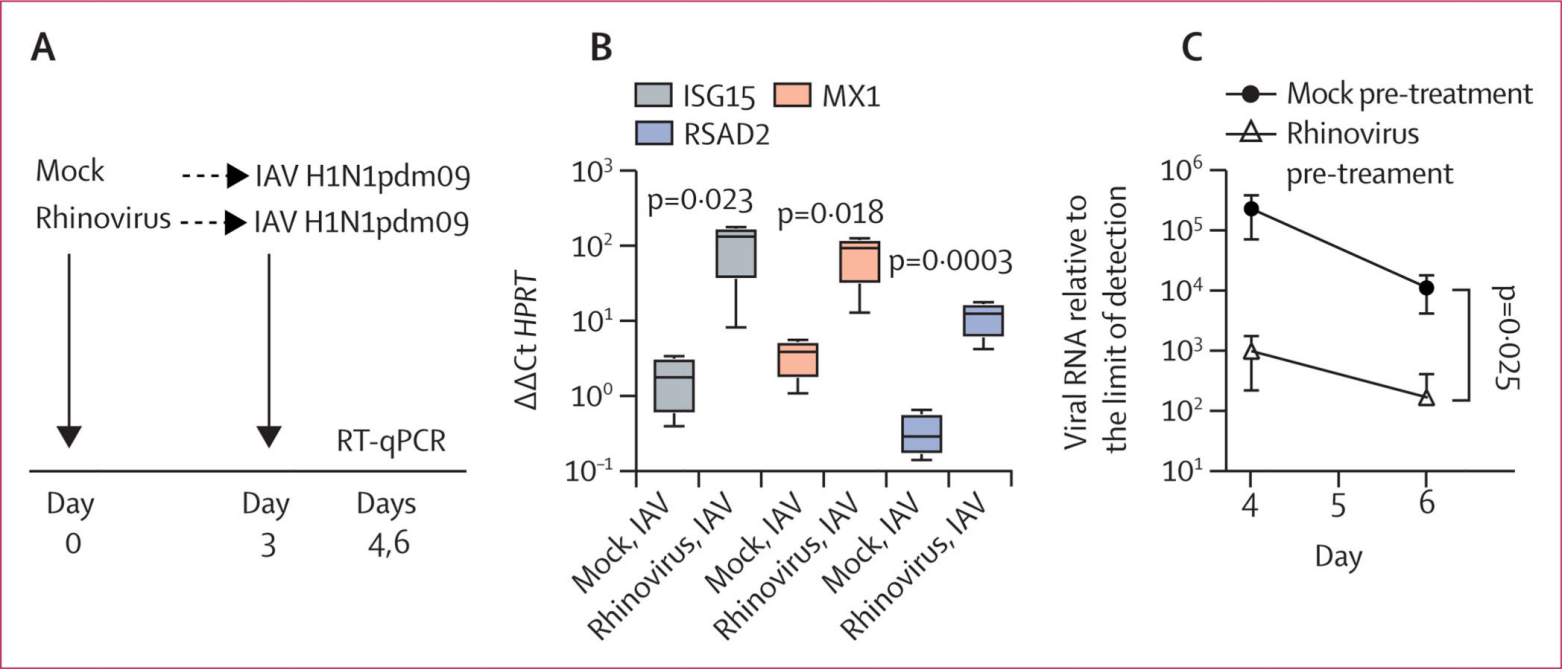
Effect of previous rhinovirus infection on epithelial gene expression and 2009 pandemic influenza A virus infection in differentiated airway epithelial cultures Data are mean (SD) of four replicates per condition. (A) Timing of sequential infections. (B) ISG mRNA expression on day 4, with or without previous rhinovirus infection. ISG expression amounts are graphed relative to the housekeeping gene *HPRT*. (C) Amount of IAV H1N1pdm09 viral RNA measured on days 4 and 6 with or without previous rhinovirus infection. The amount of viral RNA is expressed as fold change from the limit of detection. Significance of differences between mock pre-treated and rhinovirus pre-treated conditions were assessed by *t*-test (B) or two-way ANOVA (C). IAV=influenza A virus. ISG=interferon-stimulated gene. RT-qPCR=reverse-transcription quantitative PCR.

Differentiated airway epithelial cultures supported robust replication of the 2009 IAV pandemic, H1N1pdm09, with viral titres increasing more than 1000-fold after inoculation at a MOI of 0·1 (appendix p 8). Like rhinovirus, IAV H1N1pdm09 induced expression of ISGs, although the magnitude of induction was much lower than that seen during rhinovirus infection, with slight induction at 24 h and rising ISG transcript amounts by 48–72 h post-infection (appendix p 8). These data suggested that at early timepoints, ISG induction might be substantially lower during influenza infection alone compared with influenza infection plus previous rhinovirus infection. To test this hypothesis, we did a sequential infection experiment, using rhinovirus followed by H1N1pdm09 (figure 3A). Consistent with the time courses of ISG induction during each single infection, 24 h after IAV infection, there was significantly higher expression of ISG transcripts in cultures pre-infected with rhinovirus than in cultures exposed to IAV only (figure 3B). Also, as seen in sequential infection with rhinovirus and IAV PR8-GFP (figure 2), previous infection with rhinovirus led to a significant reduction in IAV H1N1pdm09 viral load at days 4 and 6 post-infection (24 and 72 h post IAV infection; figure 3C).

Similar to previous exposure with rhinovirus, interferon beta pre-treatment significantly induced ISG expression in airway epithelial cultures and reduced replication of H1N1pdm09 (appendix p 9). Consistent with previous studies, this result further indicated that the pre-activation of the antiviral interferon response can suppress IAV infection.

**Figure 4:**
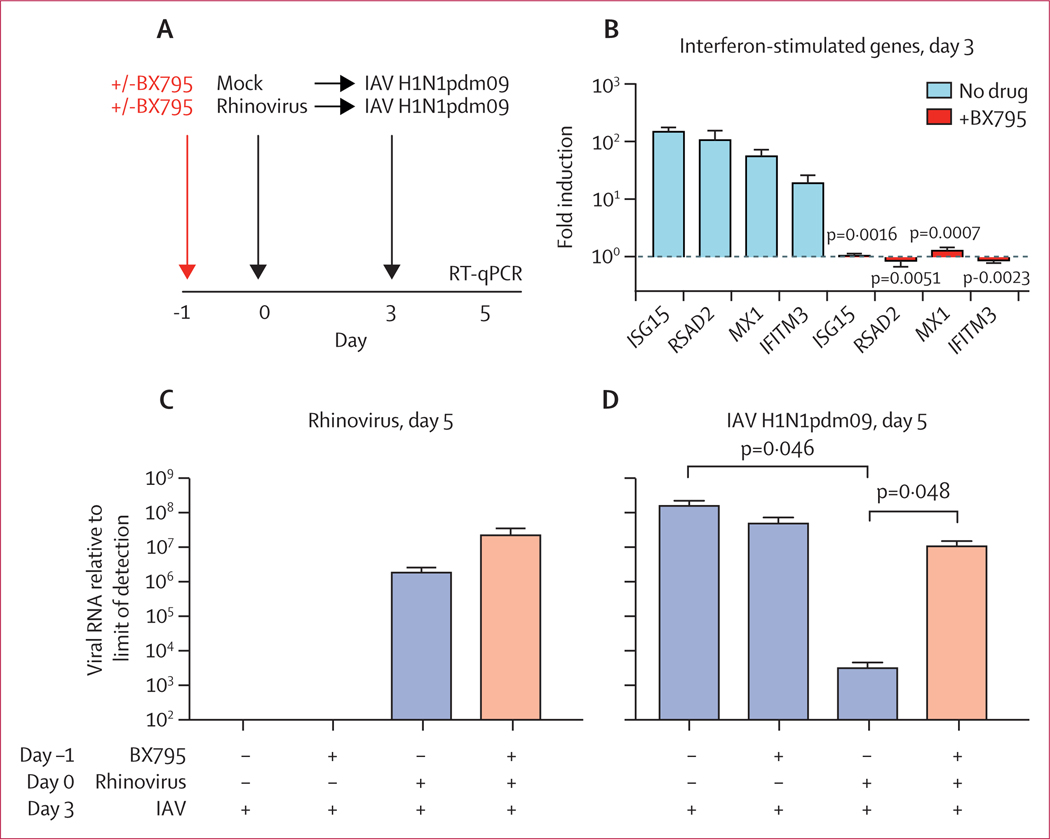
Effect of inhibiting antiviral signalling on rhinovirus–IAV interference Data are mean (SD) of four to five replicates per condition. (A) Timing of BX795 pre-treatment and sequential infections. (B) Expression of interferon-stimulated genes in rhinovirus-infected cultures on day 3, with or without BX795 pre-treatment. Bars show fold induction relative to mock treated cells. (C) Amount of rhinovirus RNA measured at day 5. (D) Amount of IAV RNA measured at day 5. The amount of viral RNA is expressed as fold change from the limit of detection. p values were calculated by *t*-test in B, C, and D. IAV=influenza A virus. RT-qPCR=reverse-transcription quantitative PCR.

As shown in figure 4A, epithelial cultures were pre-incubated with or without BX795, a drug that blocks innate immune signalling required for the interferon response,^16^ then mock infected or infected with rhinovirus and incubated for 3 days, followed by infection with IAV H1N1pdm09. RT-qPCR for ISG mRNA revealed that BX795 completely blocked induction of four ISGs previously shown to limit IAV replication (figure 4B).^19^ Cultures were then infected with IAV H1N1pdm09, and collected for viral RNA isolation and quantification by RT-qPCR on day 5 post-infection. The amount of rhinovirus RNA during rhinovirus–IAV co-infection was not significantly different in the presence of BX795, although there was a trend towards higher rhinovirus amounts in the presence of the drug (figure 4C). By contrast, BX795 pre-treatment had a marked effect on the amount of IAV RNA. Consistent with observations from 24 h and 72 h post-IAV infection (figure 3), there was a significant reduction in IAV in wells pre-infected with rhinovirus, with an approximate 50 000-fold decrease in H1N1pdm09 amounts in cultures previously infected with rhinovirus compared with wells infected with H1N1pdm09 alone (figure 4D). However, in rhinovirus-infected wells that had been pre-treated with BX795, H1N1pdm09 replication was largely restored and not significantly different than amounts in cells without rhinovirus pre-infection (figure 4D).

## Discussion

Herein, we studied the role of rhinovirus in mediating viral interference, a phenomenon in which infection with one virus alters host susceptibility to related or unrelated viruses.^1^ Our results indicate that previous infection with rhinovirus inhibits infection with influenza A virus by activating antiviral defences in the target tissue of both viruses—the human airway epithelium. Increasing use of PCR-based detection over the past decade has revealed an unexpectedly high prevalence of rhinovirus in the human airway, previously unappreciated as rhinovirus is not readily detected by previous techniques such as viral culture and immunostaining.^17,20–22^ The ability to detect rhinovirus infection led to the observation that the spread of the pandemic 2009 H1N1 influenza virus in Europe appeared to be interrupted by the autumn rhinovirus epidemic following school re-entry, raising the question of interference between these two viruses and providing the impetus for this study.^2–4^

Results of our clinical data analysis contribute to accumulating evidence for rhinovirus–IAV interference, which is largely based on comparing observed versus expected rhinovirus and IAV co-detection rates in patients. We examined an adult patient population over three winter seasons in the CT and NY area (USA) covered by the Yale-New Haven Hospital health-care system. Although our study was limited to adults aged 21 years or older, the low co-detection rates we observed for rhinovirus and IAV in this population are strikingly similar to results from different patient populations, including a study of 2121 paediatric samples from the 2009 H1N1 IAV pandemic in France; 1247 samples from children who were symptomatic in Australia from 2003; 33 652 samples from patients of all ages in Australia over 12 years; and 44 230 samples from patients of all ages in Scotland collected over 9 years.^3,8–10^ Nickbash and colleagues also presented a mathematical model further supporting viral interference.^10^ The similarities in results across different geographies, populations, and study designs support the idea that low virus co-detection rates have a biological basis rather than representing a confounder in a particular patient group. Here, we introduce a web tool for co-detection analysis and data sharing, to support this type of analysis from more geographical regions and populations.

Several mechanisms have been proposed to mediate viral interference, including direct blockade of viral entry receptors for one virus by another virus, viral competition for host cell resources, and viral induction of innate or adaptive immune responses that protect against a related or distinct virus.^1,23–25^ Studies showing that infection with rhinovirus and other RNA viruses, even when asymptomatic, can induce ISG expression in the human airway focused our attention on the antiviral interferon response as a possible mechanism.^13,26–28^ This response is initiated when viral nucleic acids are detected by innate immune sensors within infected cells, leading to production of type I and type III interferons and induction of antiviral ISGs.^29^ Interferon was first characterised by Isaacs and Lindemann in 1957, in a series of experiments showing that a substance produced by virus-exposed egg membranes could prevent influenza virus infection in naive eggs,^23^ and is now known to be an effective defence mechanism against many viruses.

We investigated rhinovirus–IAV interference using an in-vitro model of the differentiated human airway epithelium: a model in which both viruses replicated, in contrast to animal models, and in which cultures survived both infections, in contrast to cell lines. Our results show that either interferon pre-treatment or previous infection with rhinovirus suppresses IAV replication, that previous infection with rhinovirus greatly enhances ISG expression at the early stages of IAV infection, and that preventing ISG induction rescues IAV replication after rhinovirus infection (figures 2–4). These results provide strong experimental evidence that the interferon response triggered by rhinovirus infection protects the airway epithelium from IAV infection. This model also offers the possibility of further dissecting the parameters governing interference. For example, based on the kinetics of the antiviral response induced by each infection (appendix p 8), we would predict that infection with IAV before peak ISG induction by rhinovirus (eg, 2 h post-rhinovirus infection) would have less of a suppressive effect on IAV than what we observed when infecting 3 days post-rhinovirus infection.

It is important to note that our experimental model does not capture all possible mechanisms of interference in vivo. Studies in animal models have shown evidence for heterologous immunity, in which infection with one virus enhances the adaptive immune defence against another.^24^ Also, sequential infections might induce cross-protective innate immunity through additional mechanisms. A study in mice showed that alterations in mucosal innate immune responses associated with cells surviving IAV infection could mediate interference among distinct influenza strains for up to 3 weeks.^30^ One way to evaluate the importance of viral interference and its mechanisms in human populations will be further studies in humans. Our model predicts that individuals with genetic or acquired defects in the interferon-related signalling pathways will have higher rates of viral co-infection coupled with depressed local ISG expression in the airway. Longitudinal studies of viral detection and airway host response in humans will provide insight into the role of the interferon response in susceptibility to sequential viral infections in vivo.

The COVID-19 pandemic highlights the urgency of understanding and predicting the spread of respiratory viruses, to design effective interventions. Severe acute respiratory syndrome coronavirus 2 (SARS-CoV-2) transmission is expected to intersect with the annual autumn rhinovirus epidemic and the winter influenza season in 2020–21. The work presented here raises the question as to whether rhinovirus and other respiratory viruses will interfere with SARS-CoV-2. Studies indicate that like IAV and many other viruses, SARS-CoV-2 is inhibited by interferons. If interference by rhinovirus disrupted the 2009 IAV epidemic in Europe, viral interference, or even therapeutic induction of the airway interferon response, might have the potential to disrupt the current pandemic. However, more work is needed to establish the effect of rhinovirus and airway interferon responses on SARS-CoV-2, especially in light of evidence that ACE2, the viral entry receptor for SARS-CoV-2, is itself an ISG.

Finally, the results reported here suggest re-evaluating the current conception of rhinovirus infection. Rhinovirus has long been known as the most frequent cause of the common cold, but work over the past decade showed that rhinovirus is also present at unexpectedly high rates in individuals who are asymptomatic and that even asymptomatic infections can trigger ISG expression in the airway mucosa.^20–22,26–28^ Our findings suggest that although rhinovirus can be a pathogen, rhinovirus infections might also function in host protection by changing the set point of epithelial innate immunity and blocking infection by viruses of a higher pathogenicity. In fact, the annual autumn rhinovirus epidemic associated with school reentry in the northern hemisphere might be a major factor determining the timing and severity of the annual winter influenza epidemic, an important cause of morbidity and mortality every year. Together with previous work, the findings presented here indicate that viral interference should be considered in efforts to predict and design interventions for respiratory virus epidemics, including annual seasonal influenza epidemics and the ongoing COVID-19 pandemic.

## Supplementary Material

1

mmc3

mmc

mmc2

mmc1

## Figures and Tables

**Table:** Expected versus observed co-detections in virus pairs, respiratory virus PCR panel, Nov 1 to March 1, 2016–19

	Expected co-detections	Observed co-detections	Odds ratio (95% CI)	p value*
Rhinovirus–IAV	67	12	0·16 (0·09–0·28)	1·08 × 10^−12^
Rhinovirus–RSV	49	13	0·24 (0·14–0·42)	5·25 × 10^−8^
Rhinovirus–hMPV	21	7	0·31 (0·15–0·66)	0·0020
Rhinovirus–PIV	11	5	0·42 (0·17–1·01)	0·066
Rhinovirus–IBV	9	3	0·30 (0·10–0·96)	0·047
Rhinovirus–adenovirus	3	5	1·50 (0·59–3·79)	0·39
IAV–RSV	46	10	0·20 (0·11–0·37)	2·58 × 10^−8^
IAV–hMPV	20	3	0·14 (0·04–0·44)	1·43 × 10^−4^
IAV–PIV	11	2	0·17 (0·04–0·71)	0·0091
IAV–IBV	9	3	0·33 (0·10–1·03)	0·067
IAV–adenovirus	3	3	0·92 (0·29–2·98)	1
RSV–hMPV	15	1	0·06 (0·01–0·46)	3·90 × 10^−4^
RSV–PIV	8	0	0·06 (0·004–0·95)	6·41 × 10^−3^
RSV–IBV	6	1	0·15 (0·02–1·06)	0·045
RSV–adenovirus	2	1	0·40 (0·06–2·93)	0·73
hMPV–PIV	3	3	0·89 (0·28–2·79)	1·0
hMPV–IBV	3	1	0·36 (0·05–2·57)	0·53
hMPV–adenovirus	1	2	2·01 (0·49–8·33)	0·27
PIV–IBV	1	0	0·32 (0·02–5·25)	0·41
PIV–AdV	1	0	0·87 (0·05–14·21)	1·0
IBV–adenovirus	0	1	2·25 (0·31–16·43)	0·37

IAV=influenza A virus. RSV=respiratory syncytial virus. hMPV=human metapneumovirus. PIV=parainfluenza virus. IBV=influenza B virus.

* Fisher’s exact test or χ^2^ test.

## Research in context

### Evidence before this study

We searched PubMed and Web of Science for articles in any language pertaining to interference between rhinovirus and influenza A virus (IAV) from database inception up until May 10, 2020, using search terms “rhinovirus” and “viral interference” and “rhinovirus” and “interference”. To capture all possible articles showing rhinovirus-mediated interference, we did not include the term influenza. Three reports showed data suggesting that the autumn rhinovirus epidemic interrupted and delayed the spread of the 2009 H1N1 pandemic IAV in Europe. Five studies of more than 1000 respiratory samples reported lower than expected co-detection rates of rhinovirus and IAV in patients. One study showed that inoculation with a high dose of rhinovirus attenuated subsequent IAV infection in a mouse model.

### Added value of this study

We evaluated interference between rhinovirus and IAV using clinical data and an experimental model. In the first study of an adult patient population, to our knowledge, we observed a significant negative association between rhinovirus and IAV during peak co-circulation of both viruses over 3 years. We present a web tool for co-detection analysis and data sharing in other patient populations. We also introduce a new experimental model of interference using sequential infection of ex-vivo differentiated human airway epithelium. Both viruses replicate in this model, in contrast to animal models that do not support rhinovirus replication. With use of a green fluorescent protein reporter IAV and an IAV isolate from the 2009 pandemic, we showed that previous rhinovirus infection suppresses subsequent IAV infection. Furthermore, we also showed that previous rhinovirus infection induces antiviral responses in the airway epithelium and that blocking innate immune signalling in host cells restores IAV replication after rhinovirus infection. This work provides clear clinical and experimental evidence for rhinovirus interference with IAV infection and establishes a physiologically relevant model for future studies.

### Implications of all the available evidence

Together with previous observations, this work provides compelling evidence that rhinovirus infection can protect against subsequent IAV infection and supports the idea that interference from rhinovirus delayed the spread of the 2009 IAV pandemic in Europe. These findings indicate that viral interference might shape and potentially interrupt an epidemic, and should be considered when predicting and designing interventions for seasonal influenza epidemics and the ongoing COVID-19 pandemic.
