# Supplementary figures and images for: Interference between rhinovirus and influenza A virus: a clinical data analysis and experimental infection study
Source: Lancet Microbe. Author manuscript; Available in PMC 2021 Oct 1. (PMC7580833; doi:10.1016/s2666-5247(20)30114-2)

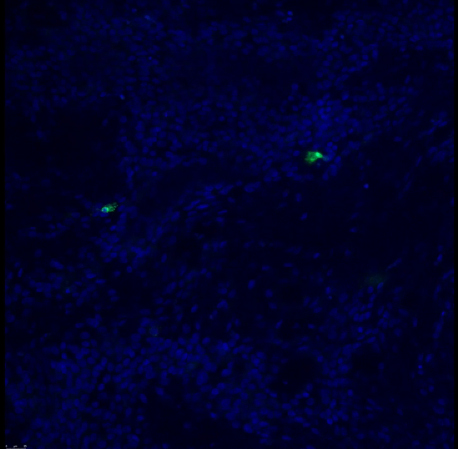

Supplement: mmc3 [file NIHMS1628631-supplement-mmc3.jpg]
