# Supplementary material for: Interference between rhinovirus and influenza A virus: a clinical data analysis and experimental infection study
Source: Lancet Microbe. Author manuscript; Available in PMC 2021 Oct 1. (PMC7580833; doi:10.1016/s2666-5247(20)30114-2)
Supplement: mmc1 [file NIHMS1628631-supplement-mmc1.pdf]

THE LANCET
Microbe

Supplementary appendix

This appendix formed part of the original submission and has been peer reviewed. We post it as supplied by the authors.

Supplement to: Wu A, Mihaylova V T, Landry M L, et al. Interference between rhinovirus and influenza A virus: a clinical data analysis and experimental infection study. *Lancet Microbe* 2020; published online September 4. https://doi.org/10.1016/S2666-5247(20)30114-2.

Supplementary Materials

Supplementary Methods

Table S1. Methods used for Influenza A diagnosis, YNHH, July 2016- June 2019

Table S2. Tests in the Yale-New Haven Hospital respiratory virus PCR panel.

Table S3. Total detections for each virus in the respiratory virus PCR panel, November 1-March 1, 2016-2019

Fig S1. Age and sex of patients undergoing multiplex testing for respiratory viruses in the Yale-New Haven Healthcare System, Nov 1-Mar 1, 2016-2019.

Fig S2. Bioinformatics pipeline and inclusion criteria for co-detection analysis.

Fig S3. Effect of rhinovirus infection three days prior or treatment with interferon β one day prior on influenza replication and interferon stimulated gene induction.

Video S1. IAV infection only. Differentiated bronchial epithelial cell cultures were mock infected, and after three days were infected with influenza GFP reporter virus. Video shows image stacks of 17M thick optical section, 24hr post IAV infection. Green indicates IAV-infected cell.

Video S2. IAV infection after rhinovirus infection. Differentiated bronchial epithelial cell cultures were infected with HRV-01A, and after three days were infected with influenza GFP reporter virus. Video shows image stacks of 17M thick optical section, 24hr post IAV infection. Green indicates IAV-infected cell.

URL for co-detection calculator webtool: https://codetection.foxmanlab.yale.edu

Supplementary Methods

Clinical Data

Test methods employed to establish seasonality

Test methods in use to detect RV or IAV at YNHH from 2016-2019 included: direct fluorescent antigen detection for IAV (SimulFluor respiratory Screen, Light Diagnostics), antigen detection for IAV by lateral flow immunoassay (BinaxNOW, Alere Diagnostics and BD Veritor System, Becton Dickinson), rapid real time PCR for IAV (Xpert Xpress Flu, Cepheid Diagnostics), and lab-developed PCR tests for RV and IAV (Tables S1, S2).

Experimental methods

Air-liquid interface culture and infection of primary human bronchial epithelial cells

Primary human bronchial epithelial cells from healthy adult donors were obtained commercially (Lonza, Walkersville, MD, USA) and cultured at air-liquid interface according to manufacturer's instructions using reduced hydrocortisone (Stem Cell Technologies, Vancouver, Canada). Cells were allowed to differentiate for four weeks by which time they displayed beating cilia and mucus production. Our preliminary experiments established that infection of these cultures with an MOI of 0·1 resulted in robust viral amplification for each virus based on qPCR for viral RNA, so this MOI was used for co-infection experiments, performed as described in the main text.

Generation of virus stocks

To generate virus stocks, Rhinovirus 1A (HRV-01A; ATCC VR-481) was cultured on H1-HeLa cells (ATCC CRL-1985). The titer of the virus stock was determined using HeLa cell plaque assay as reported previously.[1] Pandemic Influenza A virus H1N1pdm09, Strain A/California/07/2009, was obtained from (ATCC VR-1894) and influenza A virus PR8-GFP was generously shared by the Garcia-Sastre laboratory (Icahn School of Medicine at Mount Sinai, New York, NY, USA)[2]. IAV strains were propagated in embryonated eggs and the titer was measured using plaque assay on MDCK cells.

RT-qPCR and confocal microscopy

For RT-qPCR, RNA was isolated from each well of differentiated epithelial cells using the Aurum RNA mini kit (BioRad, Hercules, California) or the QIAGEN RNeasy kit by incubating each 24-well insert with 350 µl lysis buffer at room temperature for 5 minutes, followed by RNA isolation and cDNA synthesis using iScript cDNA synthesis kit (BioRad). To assess viral load and the expression of interferon stimulated genes ISG15, RSAD2 (viperin), MX1, IFITM3, and housekeeping gene HPRT, qPCR was performed using SYBR green iTaq universal (BioRad) per manufacturer's instructions. Viral RNA was quantified using primers to the RV or influenza A genome. Viral RNA per ng total RNA is graphed as fold change from the limit of detection (40 cycles of PCR) as $2^{40\text{-}Ct}$. ISG mRNA levels are graphed as fold change from mock-treated cells (Figs 2, 4, and S4) or are presented relative to the level of mRNA for the housekeeping gene HRPT ($2^{-\Delta\Delta CT}$) (Fig 3B, Fig S3).

The following primers were used:
ISG15 (F-CATCTTTGCCAGTACAGGAGC; R-GGGACACCTGGAATTCGTTG)
RSAD2 (F-TCGCTATCTCCTGTGACAGC;R-CACCACCTCCTCAGCTTTTG)
MX1 (F-AGAGAAGGTGAGAAGCTGATCC;R-TTCTTCCAGCTCCTTCTCTCTG)
IFITM3 (F-ATCGTCATCCCAGTGCTGAT;R-ATGGAAGTTGGAGTACGTGG)
HPRT (F:TGGTCAGGCAGTATAATCCAAAG; R: TTTCAAATCCAACAAAGTCTGGC)
HRV-01A (F- CAGGCCAAATTAAAGTCAATAAGC; R- AGGCTGAAGTTTGGTTTTGC)
IAV GFP-PR8 (F-ATACCCAAGCAGAAAGTGGC; R-AGCCGGTCAAAAATCACACT)
IAV H1N1pdm09 (F GGGTGGACAGGGATGGTAGA; R-TCTGTGTGCTCTTCAGGTCG)

For confocal microscopy, cultures infected with GFP expressing influenza virus were fixed with 4% formaldehyde for 10 min at room temperature, then washed three times with PBS. For nuclear staining, cells were permeabilized with 0·1% Triton for 10 minutes and rinsed 3 times with PBS, then stained with 0·25 micromolar DAPI (Invitrogen cat no· D1306) for 30 minutes at room temperature, then rinsed with PBS. For imaging, the apical chamber of each culture was filled with PBS and cultures were imaged using an inverted Zeiss LSM 880 Airyscan or a Leica SP8 inverted confocal microscope by placing the tissue culture insert directly on a glass coverslip. GFP positive cells per high power field were counted using 20x objective and 4·6µM thick optical sections and cultures were imaged as projections of 17µM thick optical sections.

Statistical Analysis

We used Python v3·7 to condense the raw clinical data into a table with number of detections of each virus and co-detections of virus pair. For each virus pair, associations were evaluated using two by two contingency tables using open source Python tools available from scipy.org v1·3·0 including odds ratios and associated 95% confidence intervals. To evaluate the significance of the difference between the observed and expected co-detections, significance testing was performed using Chi-squared or Fishers exact test ($p < 0{\cdot}05$). When any expected frequency within the contingency table was less than 5, the Fisher's exact test was used; otherwise, a Chi-squared test of independence was used. If any value in the contingency table was 0, Haldane's correction was applied for calculation of the odds ratio. The primary outcome was RV/IAV co-detections and therefore although co-detection analysis is reported for other virus pairs, the Bonferroni correction was not applied in calculating p values. Data for RV and IAV co-detections were spot-checked for accuracy using clinical virology laboratory records.

Seasons employed	Method	Test and Manufacturer
2016-17 and 2017-18	Direct fluorescent antigen detection	SimulFluor Respiratory Screen, Light Diagnostics
	Antigen detection by lateral flow immunoassay	BinaxNOW Influenza A&B Card, Alere Diagnostics
		BD Veritor System for Flu A+B antigen detection (Becton Dickinson)
2018-19	Rapid real-time RT-PCR	Xpert Xpress Flu, Cepheid

Table S1. Methods used Influenza A diagnosis, YNHH healthcare system, July 2016- June 2019
All tests were performed in the clinical laboratory according to manufacturer's instructions.
Note: Rapid PCR (Cepheid) replaced antigen detection methods as the rapid screening test in 2018-19 season.

Rhinovirus (RV)*
Influenza A and B (IAV, IBV)
Parainfluenza 1, 2, and 3 (PIV 1-3)
Respiratory syncytial virus A and B (RSV A, B)
Human metapneumovirus (hMPV)
Adenoviruses (AdV)

Table S2. Respiratory virus PCR tests in the 10-virus Yale-New Haven Hospital panel, 2016-2019. Tests were performed as described previously[3], with forward primers for RV updated in 2018-2019 according to Lu et al, 2017[4]

Date range	RV	IAV	RSV	hMPV	PIV	IBV	Adeno
**11/1/18 - 3/1/19**	364	214	219	111	50	3	16
**11/1/17 - 3/1/18**	361	273	283	109	44	88	23
**11/1/16 - 3/1/17**	264	435	183	71	60	38	9
**Total**	989	922	685	291	154	129	48

Table S3. Total detections of each virus, respiratory virus PCR panel, November 1-March 1, 2016-2019, post-filtering (n=13,707 samples)

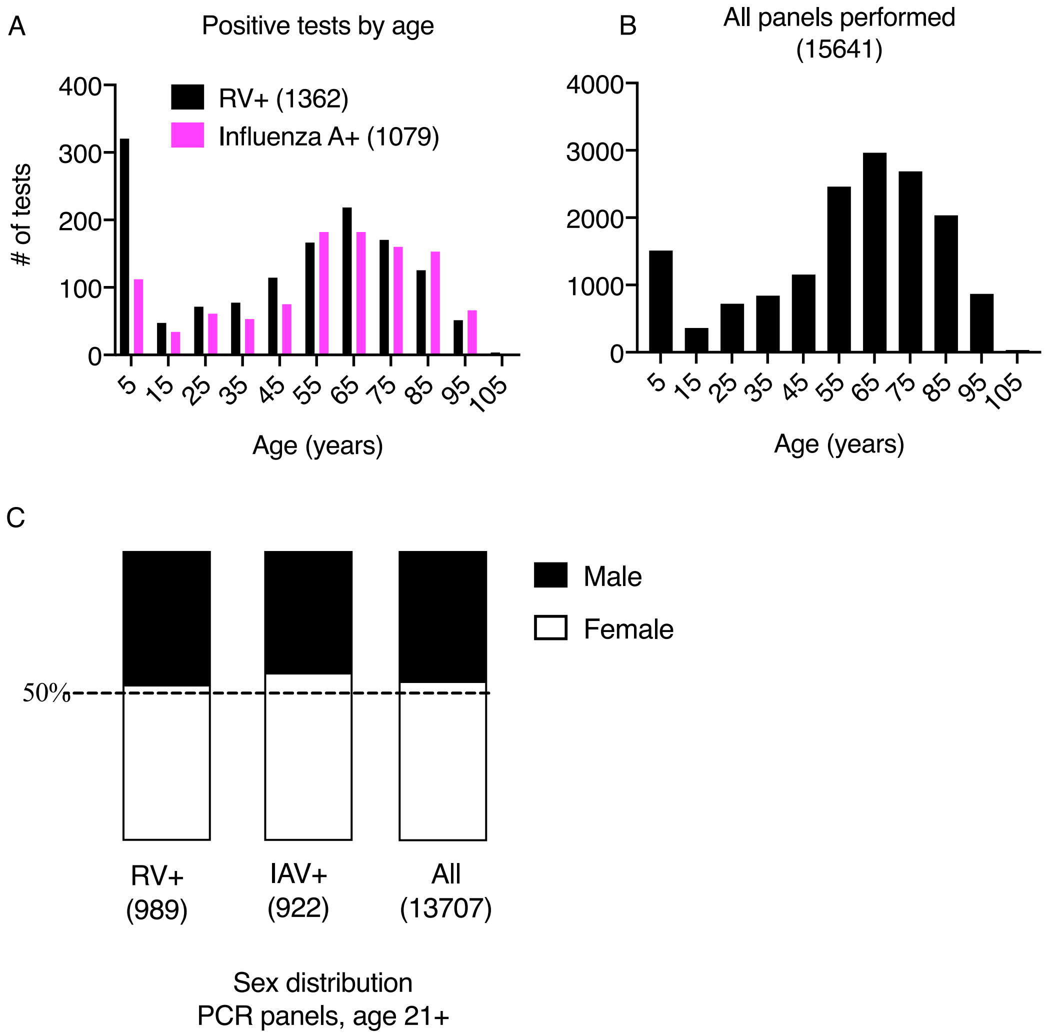

Fig S1. Age and sex of patients undergoing multiplex PCR testing for respiratory viruses Nov 1-Mar 1, 2016-2019.

(A)Age distribution of patients with positive tests for rhinovirus (black bars) or influenza A virus (pink bars) for all samples undergoing multiplex PCR testing for respiratory viruses in the YNHH healthcare system during three consecutive winter seasons, Nov 1-Mar 1, 2016-2019. (B) Age distribution of all tests performed during the same time frame. (A) and (B) include results from patients of all ages but exclude repeat tests performed on the same patient within a week (n=15,641). Bars show number of tests for each age group, labelled by bin center. (C) Breakdown by sex of 13,707 test results meeting inclusion criteria for co-detection analysis. Numbers in parentheses show total number of tests in each category.

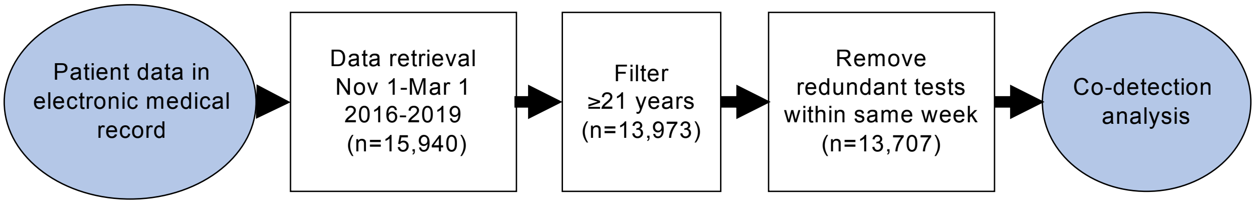

Fig S2. Bioinformatics pipeline and inclusion criteria for co-detection analysis.

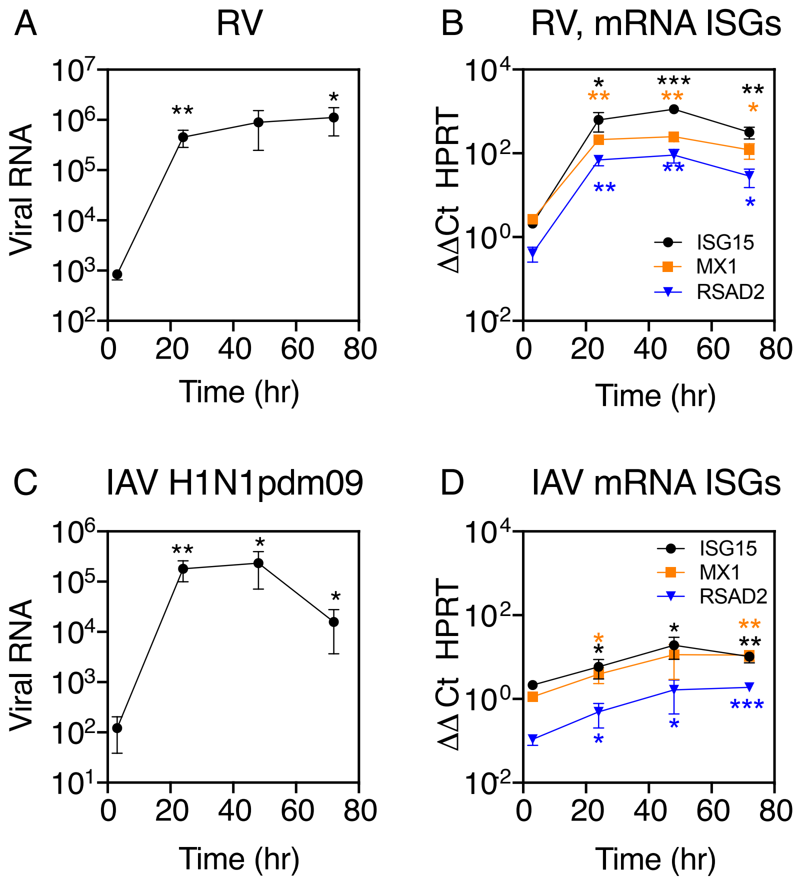

Fig S3. Time course of rhinovirus or influenza A H1N1pdm9 replication and interferon stimulated gene induction in primary human bronchial epithelial differentiated cultures.

(A-D) Differentiated cultures of human bronchial epithelial cells (HBEC) were infected with RV (HRV-01A) (A,B) or the 2009 pandemic influenza A virus (IAV_H1N1pdm09) (C,D) , incubated at 35°C, and replicate wells were collected at 3hr, 24hr, 48hr, and 72 hr for RNA isolation and RT-qPCR for viral RNA and interferon stimulated genes ISG15, MX1, and RSAD2 (Viperin). Viral growth curves (A, C) show viral RNA per ng RNA, relative to limit of detection. Host cell mRNA levels (B, D) are presented relative to the level of mRNA for the housekeeping gene HRPT ($2^{-\Delta\Delta CT}$). Mean and S.D. of 3-4 replicates per condition are shown. Asterisks indicate significant increase in the mRNA level at the indicated time point compared to the level at t=3hr for each mRNA. *p<0·05; **p<0·005 ***p<0·0001

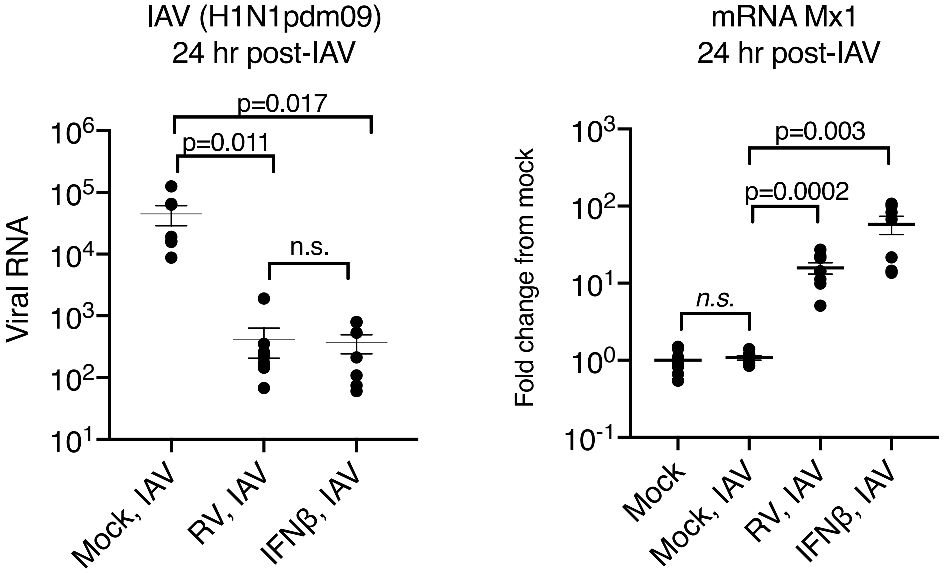

Fig S4. Effect of rhinovirus infection three days prior or treatment with interferon β one day prior on influenza replication and interferon stimulated gene induction. Differentiated primary human bronchial epithelial cells were left untreated (mock, IAV), inoculated with RV, and incubated at 35°C for 3 days (RV, IAV), or treated with 1000U/ml interferon β in the basolateral medium for 18 hr (IFNβ, IAV). Following pretreatment, cells were inoculated with IAV H1N1pdm09, then incubated for 24 hr prior to RNA isolation and RT-qPCR for viral RNA or mRNA for Mx-1. (A)Viral RNA per ng total RNA for influenza A (H1N1pdm09), graphed as fold change from limit of PCR detection. (B) Fold change in Mx1 mRNA level relative to average value of mock treated cells. Graphs show mean and S.E.M. of at 7 replicates per condition, representing combined data from two independent experiments. P-values for pairwise comparisons are shown.

Description of supplemental video files

These videos illustrate the image stacks of the 17µM thick optical sections used to generate the 3D max projections shown in Fig 2E.

Video S1. IAV infection only. Differentiated bronchial epithelial cell cultures were mock infected, and after three days were infected with influenza GFP reporter virus. Video shows image stacks of 17M thick optical section, 24hr post IAV infection. Green indicates IAV-infected cell.

Video S2. IAV infection after rhinovirus infection. Differentiated bronchial epithelial cell cultures were infected with HRV-01A, and after three days were infected with influenza GFP reporter virus. Video shows image stacks of 17M thick optical section, 24hr post IAV infection. Green indicates IAV-infected cell.

Link to co-detection calculator webtool
http://codetection.foxmanlab.yale.edu

This website allows the user to perform the statistical analysis of observed vs. expected virus co-detections described in this study by entering data in a 2x2 contingency table, with an option for data sharing by location on the world map.

References

1. Foxman EF, Storer JA, Fitzgerald ME, et al. Temperature-dependent innate defense against the common cold virus limits viral replication at warm temperature in mouse airway cells. *Proceedings of the National Academy of Sciences of the United States of America* 2015; **112**(3): 827-32.
2. Manicassamy B, Manicassamy S, Belicha-Villanueva A, Pisanelli G, Pulendran B, Garcia-Sastre A. Analysis of in vivo dynamics of influenza virus infection in mice using a GFP reporter virus. *Proceedings of the National Academy of Sciences of the United States of America* 2010; **107**(25): 11531-6.
3. Landry ML, Foxman EF. Antiviral Response in the Nasopharynx Identifies Patients With Respiratory Virus Infection. *The Journal of infectious diseases* 2018; **217**(6): 897-905.
4. Lu X, Schneider E, Jain S, et al. Rhinovirus Viremia in Patients Hospitalized With Community-Acquired Pneumonia. *The Journal of infectious diseases* 2017; **216**(9): 1104-11.
